# Succinate dehydrogenase B-deficient renal cell carcinoma with a germline variant in a Japanese patient: a case report

**DOI:** 10.1038/s41439-022-00202-z

**Published:** 2022-07-22

**Authors:** Shinichiro Higashi, Takeshi Sasaki, Katsunori Uchida, Takumi Kageyama, Makoto Ikejiri, Ryuki Matsumoto, Manabu Kato, Satoru Masui, Yuko Yoshio, Kouhei Nishikawa, Yoshinaga Okugawa, Masatoshi Watanabe, Takahiro Inoue

**Affiliations:** 1 Department of Nephro-Urologic Surgery and Andrology, Mie University Graduate School of Medicine, Mie, Japan; 2 Department of Oncologic Pathology, Mie University Graduate School of Medicine, Mie, Japan; 3 Central Laboratory, Mie University Hospital, Mie, Japan; 4 Department of Genomic Medicine, Mie University Hospital, Mie, Japan

**Keywords:** Cancer genomics, Cancer epigenetics

## Abstract

Succinate dehydrogenase (SDH)-deficient renal cell carcinoma (RCC) is a rare renal cancer. A 75-year-old Japanese female presented with gross hematuria. Computed tomography revealed two tumors in the left kidney, which were resected. Immunohistochemistry indicated negative staining for the B subunit of SDH (SDHB) in the resected specimen, leading to a final diagnosis of SDHB-deficient RCC. Genetic testing for *SDHB* showed a RCC germline variant in exon 6 (NM_003000.3:c.642 G > C) that was previously reported but associated with a novel phenotype (i.e., RCC). Twenty-six years prior, her daughter, who was 25 years old at the time, had undergone radical nephrectomy for a pathologic diagnosis of renal oncocytoma of the right kidney; SDHB immunostaining of her daughter’s tumor was also negative retrospectively. We confirmed that her daughter carried the germline variant in *SDHB* exon 6, similar to the patient. The patient had no evidence of disease progression at 15 months after surgery.

Although SDH-deficient renal cell carcinoma (RCC) occurs rarely, it was recently recognized as a unique subtype in the 2016 World Health Organization classification^1^. Most patients with SDH-deficient RCC harbor a germline variant in SDH. The estimated incidence rate of SDH-deficient RCC among all RCCs is 0.05%–0.2%^2^. Sixty cases of SDH-deficient RCC have been reported in the literature^3^. Among SDH-deficient RCCs, SDHB-deficient RCC is the predominant type, whereas SDHA-, SDHC-, and SDHD-deficient RCC are less common^4,5^.

We describe a germline variant in *SDHB* exon 6 (NM_003000.3:c.642 G > C) that was previously reported but associated with a novel phenotype (RCC). We retrospectively reviewed the patient’s daughter’s renal tumor, which was diagnosed as a renal oncocytoma 26 years prior. Negative immunostaining for SDHB in the daughter’s tumor and the same variant identified through blood samples changed the diagnosis of renal oncocytoma to SDHB-deficient RCC.

A 75-year-old Japanese female was referred to our hospital with tumors in the left kidney, which were detected during examination for gross hematuria. She had no significant previous disease or family history of paraganglioma/pheochromocytoma (PPGL), gastrointestinal stromal tumor, or pituitary adenoma. Twenty-six years previous, her daughter, who was 25 years old, had undergone radical nephrectomy of the right kidney, with a pathologic diagnosis of renal oncocytoma. Enhanced computed tomography (CT) revealed a 3.8 × 2.8 cm tumor in the upper pole and a 1.2 × 1.1 cm tumor in the lower pole regions of the left kidney of our patient (Fig. 1A, B). There was no obvious metastasis on systemic radiologic investigations. All blood examinations were within normal limits. We performed robot-assisted partial nephrectomy, and histopathological examination revealed SDH-deficient RCC with Fuhrman grade 2/International Society of Urological Pathology grade 2 (Fig. 1C). The cells were intermediate to large in size, with cytoplasmic vacuoles containing eosinophilic fluid. Nuclei were round, and prominent nucleoli and apparent perinuclear halos were absent. Immunostaining for the B subunit of SDH (SDHB) (Abcam ab4714, clone 21A11AE7, Cambridge, UK) was negative, whereas immunostaining for scattered inflammatory cells was positive (Fig. 1C). Immunostaining for the A subunit of SDH (SDHA) (Abcam ab14715, clone 2E3GC12FB2AE2, Cambridge, UK) was positive (Fig. 1C). In addition, we performed a genetic test for *SDHB* after obtaining written consent from the patient. DNA was extracted from her renal tumor tissue, normal kidney tissue, and blood using a DNeasy blood and tissue kit (Qiagen, Valencia, CA). The primer pairs used for exon amplification (exons 1 to 8) of both the tumor and normal tissue and DNA sequencing with a 3730XL DNA analyzer (Thermo Fisher Scientific, MA USA) were performed as previously reported^6^. We used the nucleotide sequence database of *SDHB* (https://www.ncbi.nlm.nih.gov/nuccore/NG_012340.1) as a normal control. The results showed that the patient carried an RCC germline variant in *SDHB* exon 6 (NM_003000.3:c.642 G > C) (Fig. 2A, B, and C). There was no evidence of disease progression at 15 months after surgery. We retrospectively reviewed the patient’s daughter’s renal tumor, which consisted of eosinophils and oncocytes with multiple cytoplasmic vacuoles; immunohistochemical staining of SDHB in the tumor lesion was negative (Fig. 1D). Because the patient’s daughter was strongly suspected of being an *SDHB* variant carrier, the daughter underwent familial genetic testing of blood after clinical genetic counseling and was found to carry the same variant in *SDHB* exon 6 (Fig. 2D).

**Fig. 1:**
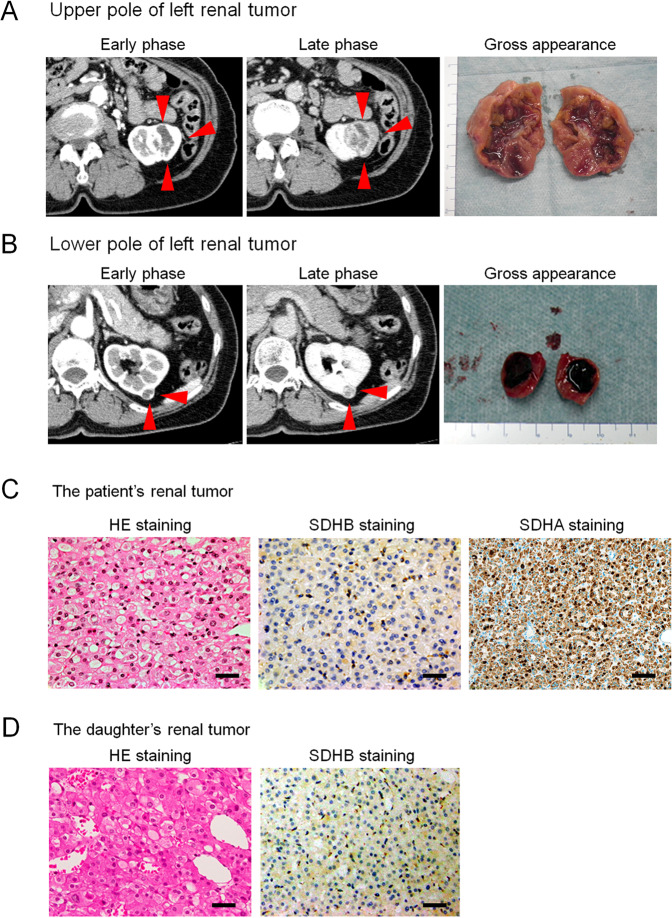
Abdominal computed tomography, gross appearance, and microscopic findings. Enhanced computed tomography (CT) showing an enlarged 3.8 × 2.8 cm tumor in the upper pole region of the left kidney and a 1.2 × 1.1 cm (arrow) (**A**) tumor in the lower pole region of the left kidney (arrow) (**B**). The tumors were well circumscribed with tan-brown (**A**) and reddish-brown (**B**) cut surfaces. Histopathological examination revealed SDH-deficient RCC with Fuhrman grade 2/International Society of Urological Pathology grade 2 (**C**). Cells were intermediate to large in size with cytoplasmic vacuoles containing eosinophilic fluid. Nuclei were round with prominent nucleoli, and apparent perinuclear halos were absent. Immunostaining for SDHB was negative but positive for scattered inflammatory cells (**C**). Immunostaining for SDHA was positive (**C**). Histopathology of the daughter’s tumors showed eosinophils and oncocytes with multiple cytoplasmic vacuoles and negative immunostaining for SDHB (**D**) (bar = 50 µm).

**Fig. 2:**
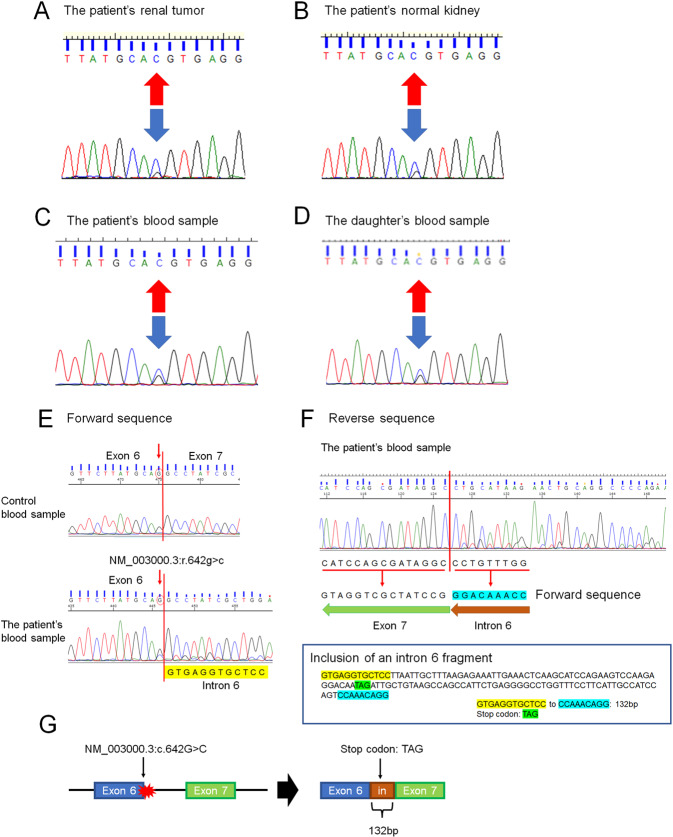
PCR-directed sequencing of *SDHB* exon 6. DNA from the patient’s tumor tissue showed a variant in RCC (NM_003000.3:c.642 G > C) (arrow) previously reported but associated with a novel phenotype (RCC) (**A**). DNA from the patient’s normal tissue and blood sample showed the same variant (NM_003000.3:c.642 G > C) (arrow) (**B** and **C**). DNA from the daughter’s blood sample showed the same variant (NM_003000.3:c.642 G > C) (arrow) (**D**). Effect of the *SDHB* variant (NM_003000.3:c.642 G > C) on splicing by RNA analysis. Forward sequence data of cloned RT–PCR products revealed that the 3′ splice site of *SDHB* exon 6 was not recognized, with aberrant transcription continuing into intron 6 (**E**). Reverse sequence data of cloned RT–PCR products revealed the presence of 132 base pairs of intronic sequences adjacent to exon 7, indicating intron 6 retention (**F**). The intronic sequence included a stop codon (TAG) (**F**). The *SDHB* variant (NM_003000.3:c.642 G > C) results in usage of the intronic splice site, leading to the inclusion of an intron fragment (132 base pairs) (NM_003000.3: r.[642 g > c;642_643ins642 + 1_642 + 132]) including a stop codon (TAG) (**G**), which may be a protein-truncating variant (NM_003000.3:p.Gln214delinsHisValArgCysSerLeuIleAlaLeuArgGluIleGluThrGlnAlaSerArgSerProArgGlyGlnTer).

Previous studies have summarized the distribution of *SDHB* germline variants in RCC^5,7^; however, the variant identified in our study has not been previously reported in RCC. Notably, the same *SDHB* variant (NM_003000.3:c.642 G > C) has been reported in patients with malignant paraganglioma^8^. Based on the ClinVar database (https://www.ncbi.nlm.nih.gov/clinvar), the pathogenic meaning of this *SDHB* variant is uncertain. Nevertheless, according to the OncoKB database (https://www.oncokb.org), the amino acid change is located in the 4Fe-4S cluster domain, which binds iron-sulfur clusters. Saxena et al. summarized missense *SDHB* variants that cause familial cancer syndromes and indicated that the 4Fe–4S cluster domain is the most common site of missense variants^9^. Moreover, as the patient’s variant involved the last nucleotide of exon 6 of the *SDHB* coding sequence, we investigated the effect of this *SDHB* variant on splicing by RNA analysis. We performed RNA extraction of blood samples and generated cDNA. cDNA PCR amplification was carried out using the primers forward (Fw) primer 5′-CAGACAAGGCTGGAGACAAACC-3′ and reverse (Rv) primer 5′-GCAATAGCTTTCCCTGGATTCAGAC-3′ for targeting *SDHB* exon 3 to exon 8. The cloned RT–PCR products were analyzed by direct sequencing, revealing that the 3′ splice site of *SDHB* exon 6 was not recognized, with aberrant transcription continuing into intron 6 (Fig. 2E). Reverse sequence data of the cloned RT–PCR products showed the presence of 132 base pairs of intronic sequence adjacent to exon 7, indicating retention of intron 6 (Fig. 2F). The intronic sequence includes a stop codon (TAG) (Fig. 2F). Thus, the *SDHB* variant (NM_003000.3:c.642 G > C) results in usage of the intronic splice site, leading to the inclusion of an intron fragment (132 base pairs) (NM_003000.3: r.[642 G > C;642_643ins642 + 1_642 + 132]) (Fig. 2G), which may constitute a protein-truncating variant (NM_003000.3:p.Gln214delinsHisValArgCysSerLeuIleAlaLeuArgGluIleGluThrGlnAlaSerArgSerProArgGlyGlnTer). Based on our findings, the patient’s and her daughter’s variant (NM_003000.3:c.642 G > C) could be a pathogenic change.

SDH is well recognized as a tumor suppressor. When double-hit inactivation occurs in *SDHx* (typically with one germline and somatic variant), the entire complex becomes unstable, resulting in degradation of the B subunit. Andrews et al. found that the risk of developing a renal tumor by the age of 60 years in *SDHB* variant carriers was 4.2% (95% CI 0.46–7.8%) by Kaplan–Meier analysis of nonprobands and 4.71% (95% CI 1.65–7.7%) by Kaplan–Meier analysis of all *SDHB* variant carriers^10^. SDH-deficient RCC generally occurs in young adulthood (i.e., median 36.8 years [range, 14–76 years])^11^, though the optimal initial evaluation and follow-up of asymptomatic carriers of *SDHx* variants have not yet been agreed upon. Nonetheless, germline mutations in *SDHx* genes are responsible for approximately 20% of cases of PPGL and are also associated with the presence of other *SDHx*-related tumors^12^. Thus, PPGL evaluation may have positive effects on outcomes, including survival. The risk of RCC is smaller than that of PPGL for *SDHx* gene variant carriers, and therefore, abdominal imaging incorporating renal imaging for RCC evaluation may be sufficient for carriers^12^.

The morphology of SDHB-deficient RCC overlaps with various patterns of histological subtypes, including chromophobe RCC, clear cell RCC, papillary RCC, sarcomatoid RCC, unclassified RCC, and renal oncocytoma^13^. Both oncocytoma and SDHB-deficient RCC show tumor cells with uniformly round nuclei and eosinophilic cytoplasm and are arranged in solid nests, acini, tubules, or a cystic pattern^11^. Nonetheless, intratumoral mast cells and cytoplastic vacuoles are rarely seen in oncocytoma, and the cytoplasm of SDHB-deficient RCC is flocculent but not truly oncocytic in nature^11^. In the case of the existence of a family history, renal oncocytoma in young adulthood should be differentiated from SDH-deficient RCC.

Although SDHB-deficient RCC shows a strong correlation with germline SDH variants, immunohistochemistry is a powerful tool to determine a patient’s phenotype, as opposed to genetic testing. Immunohistochemically, tumor cells in SDHB-, SDHC-, and SDHD-deficient RCC are negative for SDHB but positive for SDHA. Loss of SDHB staining indicates disruption of SDH complex II^2^. In contrast, tumor cells are negative for both SDHA and SDHB in SDHA-deficient RCC^1^. The reason why the SDHA protein remains stable in the presence of SDHB, SDHC, or SDHD variants is unknown^1^.

In this case, we did not have any information about an additional *SDHB* hit for SDHB deficiency. According to Knudson’s two-hit model hypothesis, loss of heterozygosity (LOH) or copy number change, deep intronic change disrupting another SDHB allele, or epigenetic dysregulation in the tumor may have occurred during tumorigenesis in this patient. Moreover, the mechanisms of differential onset of SDH-deficient RCC in the patient and her daughter remain unclear.

## HGV database

The relevant data from this Data Report are hosted at the Human Genome Variation Database at 10.6084/m9.figshare.hgv.3204.
